# Efficacy of left subclavian artery laser *in situ* fenestration combined with hybrid arch debranching surgery for aortic arch reconstruction in patients with Stanford type A aortic dissection

**DOI:** 10.3389/fcvm.2025.1622468

**Published:** 2025-11-06

**Authors:** Qi Zhang, Hao Zhao, Yongqiang Yue, Shuai Zhang, Likun Sun, Peng Xu, Chao Liu, Zhaohui Hua, Zhen Li

**Affiliations:** 1 Department of Endovascular Surgery, The First Affiliated Hospital of Zhengzhou University, Zhengzhou, China; 2 Department of Cardiovascular Surgery, The First Affiliated Hospital of Zhengzhou University, Zhengzhou, China

**Keywords:** dissecting aneurysm, aortic dissection, thoracic aorta, left subclavian artery, debranching hybrid surgery, *in situ* laser fenestration

## Abstract

**Objective:**

To investigate the early and mid-term outcomes of *in situ* laser fenestration (ISLF) of the left subclavian artery (LSA) combined with hybrid aortic arch debranching for aortic arch reconstruction in Stanford type A aortic dissection.

**Methods:**

This retrospective study analyzed 57 patients (60+ years) treated from 2018 to 2023. LSA reconstruction-related complications were defined as: anastomotic bleeding, LSA occlusion, stent migration, or fenestration-related endoleak. Patients were divided into ISLF + debranching (*n* = 29) and debranching-only (*n* = 28) groups. Outcomes were compared using t-tests and Kaplan–Meier analysis.

**Results:**

The ISLF group had shorter operative time (323.1 ± 10.3 vs. 329.4 ± 7.2 min, *P* = 0.009) and higher LSA reconstruction success (100% vs. 75%, *P* = 0.013). LSA complication rates were lower in the ISLF group (3.4% vs. 28.6%, *P* = 0.025). Five-year survival was similar (79.3% vs. 75.0%, *P* = 0.575).

**Conclusion:**

ISLF with hybrid debranching improves LSA reconstruction success and reduces complications without affecting survival.

## 1 Introduction

Open surgery is currently the preferred treatment for Stanford type A aortic dissection (1). In recent years, modified aortic arch debranching hybrid surgery based on the traditional Sun's procedure has become a research hotspot. This technique avoids deep hypothermic circulatory arrest, shortens operative and cardiopulmonary bypass (CPB) times, and reduces neurological complications (2–4). However, dissection and reconstruction of the three branches of the aortic arch, especially the left subclavian artery (LSA), remain challenging (5). In some patients, the LSA may be displaced superiorly or posteriorly due to compression by the aneurysm, or there may be congenital anatomic variations or tissue adhesions, which can increase the difficulty of reconstruction and the risk of anastomotic bleeding (6, 7). *In situ* laser fenestration (ISLF) offers a potential solution, but its efficacy compared to standard approaches remains unclear. This study evaluates whether ISLF combined with hybrid arch debranching improves LSA reconstruction outcomes in TAAD patients, with rigorous predefined endpoints addressing limitations of previous reports.

## 2 Materials and methods

### 2.1 Study population

This study is a retrospective cohort analysis. We retrospectively analyzed data from 57 patients diagnosed with Stanford type A aortic dissection who underwent debranching hybrid surgery to reconstruct the aortic arch in our Department of Endovascular Surgery from January 2018 to December 2023. Patients with incomplete clinical data or those with LSA dissection involving the distal part of the vertebral artery origin were excluded from this study. The study was approved by the local ethics committee (2023-KY-0072-002), and included patients provided written informed consent.

### 2.2 Surgical methods

#### 2.2.1 Selection of surgical approach

In the early stages of this study, patients underwent simple debranching hybrid surgery for aortic arch reconstruction. If LSA reconstruction was difficult, the LSA was ligated. To improve the success rate of LSA reconstruction, our center began using *in situ* laser fenestration of the LSA combined with debranching hybrid surgery in February 2019. Obese patients are likely to benefit from this technique because the LSA is often deeper and harder to expose and anastomose in obese patients; therefore, we prioritized *in situ* laser fenestration of the LSA for patients with a body mass index (BMI) ≥ 28 kg/m^2^. However, because this technique increases treatment costs, we communicated with the families of patients who were preoperatively predicted to have difficult LSA reconstruction, especially obese patients, and decided whether to perform *in situ* laser fenestration based on the family's wishes.

#### 2.2.2 Surgical indications and contraindications

All patients were aged 60 years or older and were diagnosed with Stanford type A aortic dissection requiring aortic arch reconstruction by preoperative aortic CT angiography. Patients with hereditary connective tissue diseases, autoimmune diseases, vasculitis, combined severe preoperative hepatic and renal dysfunction, neurological complications such as paraplegia, cerebral hemorrhage, and massive cerebral infarction, or ischemic necrosis of the lower limbs or internal organs before surgery were excluded from the study. Specific contraindications for the combined approach include: Complex dissection involving the distal vertebral artery origin (due to risk of fenestration failure and endoleak). Long-segment LSA dissection with persistent false lumen flow (increased risk of Type II endoleak). Severe tortuosity or sharp angulation (<30°) between LSA and aortic arch (technical difficulty in sheath positioning).

#### 2.2.3 Surgical procedure

Each patient was placed in a supine position on a digital subtraction angiography hybrid operating table. A median sternotomy was performed, and the sternum was split longitudinally. The innominate artery and left common carotid artery were dissected. The right axillary artery and one femoral artery were cannulated for arterial perfusion and the right atrium was cannulated for venous drainage. A left ventricular vent was placed through the right superior pulmonary vein to establish cardiopulmonary bypass (CPB). Under mild hypothermic CPB, the proximal and distal ends of a four-branched artificial graft were anastomosed to the proximal and distal ascending aorta. Depending on the intraoperative findings, root procedures such as coronary artery bypass grafting, the Bentall procedure, or the David procedure were performed. The distal anastomosis of the four-branched graft was placed more than 2 cm from the distal end of the graft and a radiopaque marker was placed. The branches of the graft were sequentially anastomosed to the innominate artery and left common carotid artery. In the uncombined surgery group, LSA bypass was performed simultaneously. A Landquist super-stiff guidewire was introduced through the femoral artery incision into the ascending aorta and a thoracic aortic stent graft was introduced over the guidewire. The stent graft size was selected on the basis of the diameter of the four-branched graft and the descending aortic diameter, with a stent diameter 10%–20% larger than the vessel diameter. Depending on the measured aortic diameter, a straight or tapered stent was chosen, and a restrictive stent was implanted distally if necessary. The proximal end of the stent graft was positioned distal to the distal end of the four-branched graft based on the radiopaque marker, and 1–2 thoracic aortic stent grafts were selected depending on the extent of the lesion.

**Figure 1:**
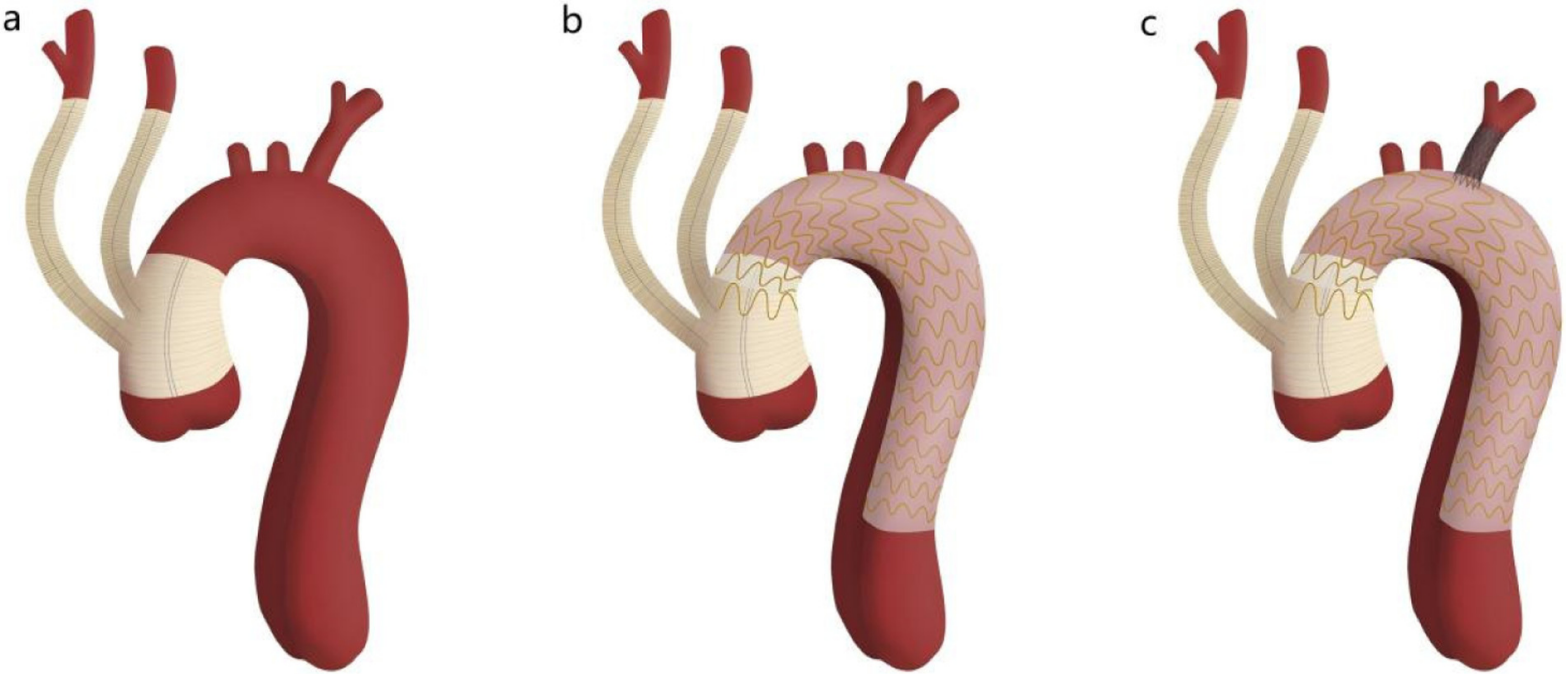
Schematic diagram of *in situ* laser fenestration of the left subclavian artery combined with the aortic arch debranching technique. Panel **a** shows the proximal and distal ends of the four-branched graft anastomosed to the proximal and distal ascending aorta, with the branch vessels anastomosed to the innominate artery and left common carotid artery. Panel **b** shows the thoracic aortic stent graft implanted distal to the distal end of the four-branched graft. Panel **c** shows *in situ* laser reconstruction of the left subclavian artery.

**Figure 2:**
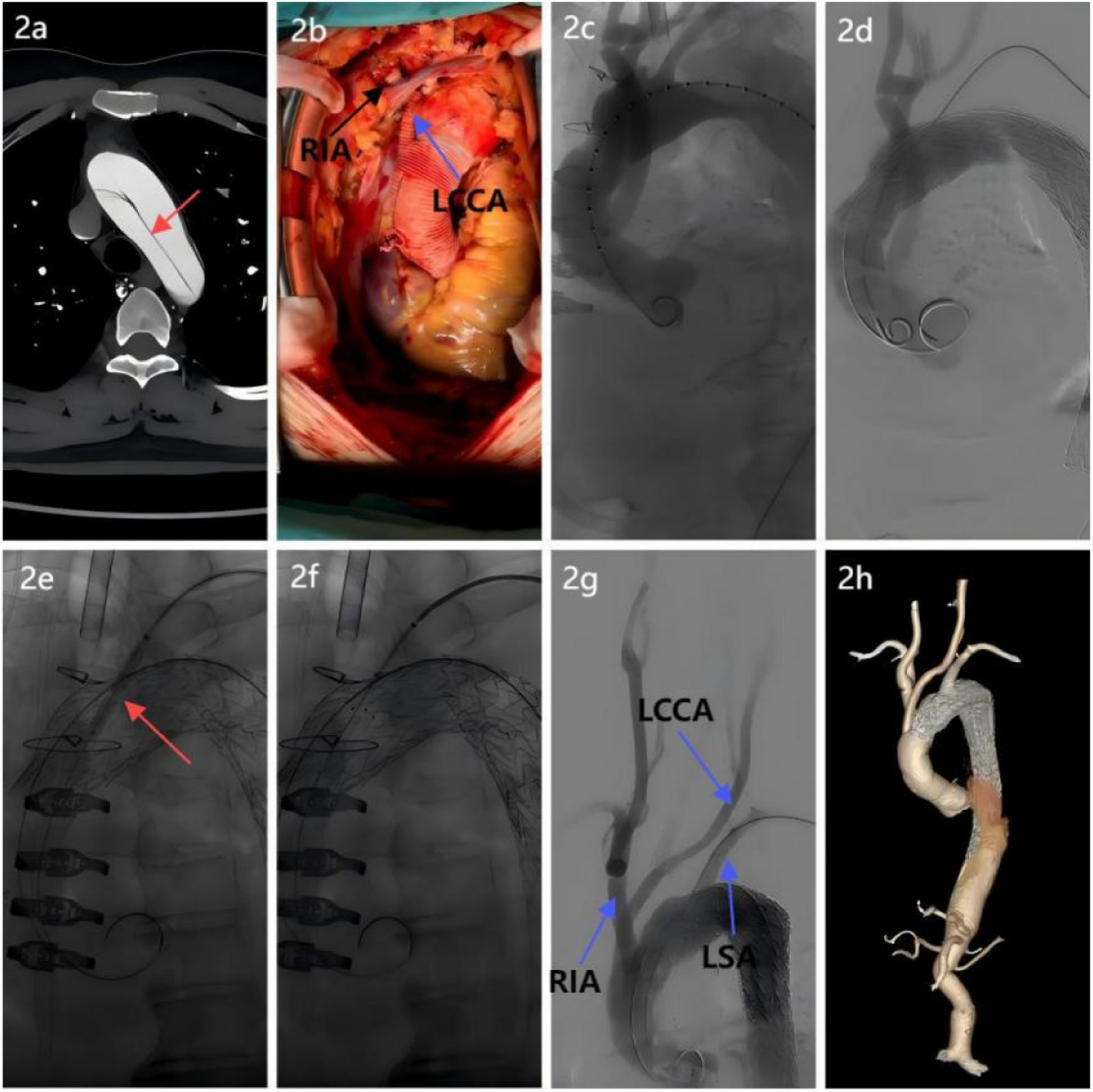
Surgical procedure breakdown. **(a)** Aortic CTA revealed an aortic arch dissection, necessitating reconstruction of the arch branch vessels; **(b)** A four-branched prosthetic graft was used: proximal and distal ends were anastomosed end-to-end with the ascending aorta. Two branches were anastomosed to the right innominate artery and left common carotid artery to restore cerebral perfusion; **(c)** Intraoperative angiography verification confirmed patency of the prosthetic graft and branches, with no anastomotic leaks; **(d)** A covered stent was implanted distal to the four-branched graft to exclude the false lumen and seal the dissection; **(e)** After laser fenestration of the left subclavian artery, a balloon was introduced, and balloon dilation revealed a significant notch; **(f)** A “parachute” technique was used to ensure proper stent apposition; **(g)** Postprocedural aortography confirmed the patency of all three supra-aortic branches with no evidence of endoleak; **(h)** Follow-up postoperative CTA of the entire aorta demonstrated patency of the three arch branch vessels, satisfactory stent morphology and position, and absence of endoleak.

In the combined surgery group, a 6 F (1 F≈0.33 mm) long sheath was introduced through the left brachial artery (puncture or cutdown). A J-shaped curved sheath (Cook Medical, USA) was most commonly used; however, if the LSA angle was sharp, a steerable sheath (Lifetech Scientific, CHN) or multi-purpose catheter (Medtronic, USA) was used. The sheath tip was positioned against the aortic stent graft, both of which were kept as perpendicular as possible. Multi-angle angiography was performed to confirm the position of the sheath tip relative to the aortic stent graft. A laser fiber was placed inside a 0.035-inch (1 inch = 25.4 mm) system 3-mm×40-mm balloon (eV3, USA), with the fiber tip extending approximately 1 cm beyond the balloon tip. The fiber and balloon were fixed relative to each other using a Y-valve. The fiber was introduced until its tip was close to the aortic stent graft. At this point, gentle pressure on the fiber caused the aortic stent graft to indent. The fiber position was kept fixed, and the laser was activated to create a fenestration, during which the surgeon felt a “falling sensation”. An assistant kept the balloon and sheath positions fixed while the fiber was gently withdrawn. A J-shaped 260 cm long “glide” wire was introduced over the balloon, and the wire usually passed through the fenestration into the aorta. Multi-angle fluoroscopy confirmed the wire position, and the balloon was advanced. Balloon expansion showed a clear “waist,” confirming successful fenestration. Depending on the measured LSA diameter, various balloon sizes were used to sequentially expand the fenestration until an LSA stent (Bard, USA) could be introduced. The stent length was usually 4 cm, and the stent was deployed using a “parachute” technique, followed by post-dilation (Figures 1,2). We used a 400-μm core diameter circular laser fiber and the VELAS30B semiconductor laser therapy device (Wuhan Boji Century Technology Co., Ltd., China) with a power of 18.0 W.

### 2.3 Patient data and follow-up

We collected baseline patient data including gender, age, BMI, comorbidities, and left ventricular ejection fraction. Operative data, including operative time, LSA reconstruction time, and surgical complications, were recorded. Patients were followed up by phone 1 month postoperatively and underwent ultrasound and aortic CT angiography at 3, 6, and 12 months postoperatively. Thereafter, patients were advised to undergo outpatient follow-up every 12 months, supplemented by phone follow-ups. The last follow-up was conducted by September 30, 2024.

### 2.4 Statistical methods

Statistical analysis was performed using SPSS 26.0 software (IBM, Chicago, IL, USA). Quantitative data are expressed as mean ± standard deviation (± s) and group comparisons were made using independent sample t-tests. Categorical data are expressed as frequency (percentage) and group comparisons were made using chi-square tests. Kaplan–Meier curves and Log-rank tests were used for survival analysis. A *P*-value <0.05 was considered statistically significant.

## 3 Results

The study population included 37 men and 20 women, with an average age of 67.9 ± 4.1 years (range: 60.8–77.5 years). Among them, 29 patients underwent *in situ* laser fenestration of the LSA combined with debranching hybrid surgery (combined surgery group), including 18 male and 11 female patients, with an average age of 67.8 ± 4.5 years (range: 60.8–77.5 years). The remaining 28 patients underwent simple debranching hybrid surgery (uncombined surgery group), including 19 male and 9 female patients, with an average age of 67.3 ± 3.6 years (range: 61.5–75.5 years).

There were no statistically significant differences between the two groups in terms of gender, age, hypertension, diabetes, renal insufficiency, moderate to severe aortic valve regurgitation, left ventricular ejection fraction, or BMI (all *P* > 0.05, Table 1).

**Table 1:** Comparison of baseline characteristics in patients with Stanford type A aortic dissection.

Variable	Overall data		*p* value
	CSG (*n* = 29)	USG (*n* = 28)	
Demographic			
Age (y, mea*n* ± SD)	67.8 ± 4.5	67.3 ± 3.6	0.093
BMI (kg/m^2^, mean ± SD)	27.83 ± 1.94	27.29 ± 2.01	0.304
Male (no.)	18 (62.1)	19 (67.9)	0.647
Cormorbidity			
Diabetes (no.)	13 (2.0)	17 (11.3)	0.230
Renal insufficiency (no.)	6 (20.7)	6 (21.4)	0.945
Moderate to severe aortic valve Regurgitation (no.)	6 (20.7)	8 (28.6)	0.490
COPD (no.)	5 (17.2)	4 (14.3)	1.000
CAD (no.)	6 (20.7)	6 (21.4)	0.945
Stroke (no.)	10 (34.5)	9 (32.1)	0.851
Hyperlipidemia (no.)	19 (65.5)	17 (60.7)	0.707
Smoking (no.)	14 (48.3)	12 (42.9)	0.681
LVEF (%, mean ± SD)	58.5 ± 6.6	56.4 ± 6.3	0.228

CSG, combined surgery group; USG, uncombined surgery group; BMI, body mass index; COPD, chronic obstructive pulmonary disease; CAD, coronary artery disease; LVEF, left ventricular ejection fraction.

There were no statistically significant differences between the two groups in terms of root reconstruction techniques or CPB time (*P* = 0.323). Compared with the uncombined surgery group, the combined surgery group had significantly shorter operative time and LSA reconstruction time (*P* < 0.001). The LSA reconstruction rate was higher in the combined surgery group than in the uncombined surgery group (*P* = 0.0045, Table 2).

**Table 2:** Comparison of operative data between the combined surgery group and the uncombined surgery group in patients with Stanford type A aortic dissection.

Variable	Overall data		*p* value
	CSG (*n* = 29)	USG (*n* = 28)	
Demographic			
Simple ascending aortic replacement (no.)	15 (51.7)	16 (57.1)	0.681
Bentall + Ascending aortic replacement (no.)	8 (27.6)	7 (25.0)	0.825
David procedure + Ascending aortic replacement (no.)	6 (20.7)	5 (17.9)	0.786
Coronary artery bypass grafting (no.)	6 (20.7)	8 (28.6)	0.490
Cormorbidity			
CPB time (mean ± SD, min)	136.2 ± 5.7	137.7 ± 5.4	0.323
Operative time (mean ± SD, min)	323.1 ± 10.3	329.4 ± 7.2	0.009
LSA reconstruction time (mean ± SD, min)	32.1 ± 2.8	43.2 ± 6.7	<0.01
LSA reconstruction (no.)	29 (29/29)	21 (75.0)	0.013

CSG, combined surgery group; USG, uncombined surgery group; CPB, cardiopulmonary bypass; LSA, left subclavian artery.

There were no statistically significant differences between the two groups in the rates of pulmonary infection, unplanned reoperation, continuous renal replacement therapy, transient neurological dysfunction, or in-hospital mortality (all *P* > 0.05). None of the patients in either group experienced complications such as cerebral hemorrhage, permanent paraplegia, endoleak, or posterior circulation ischemia. Compared with the uncombined surgery group, the combined surgery group had significantly lower rates of LSA reconstruction-related complications and recurrent laryngeal nerve injury (*P* = 0.025, Table 3).

**Table 3:** Comparison of postoperative complications between the combined surgery group and the uncombined surgery group in patients with Stanford type A aortic dissection.

Variable	Overall data		*p* value
	CSG (*n* = 29)	USG (*n* = 28)	
Demographic			
In-hospital mortality	2 (6.9)	2 (7.1)	1
Pulmonary infection	5 (17.2)	8 (28.6)	0.308
Unplanned reoperation	1 (3.4)	4 (14.3)	0.328
CRRT	2 (6.9)	1 (3.6)	1
New cerebral infarction	2 (6.9)	3 (10.7)	0.967
Transient neurological dysfunction	2 (6.9)	1 (3.6)	1
LSA reconstruction-related complications	1 (3.4)	8 (28.6)	0.025
Recurrent laryngeal nerve injury	1 (3.4)	7 (25.0)	0.013
Left upper limb weakness	0 (0.0)	3(10.7)	0.223

CSG, combined surgery group; USG, uncombined surgery group; CRRT, continuous renal replacement therapy; LSA, left subclavian artery.

Postoperative CT angiography revealed patent false lumen in the distal aorta (distal to stent graft) in % (24/29) of the CSG group vs. % (25/28) of the USG group (*P* = 0.812). No correlation was found between false lumen patency and LSA fenestration/stent placement (*P* = 0.706).

## 4 Patient outcomes and follow-up

The follow-up time was 56.3 ± 2.8 months (range: 0–67.1 months). In the combined surgery group, three patients were lost to follow-up, with a follow-up rate of 89.7%. Among the remaining patients, three died within 5 years, including one who died 2 days postoperatively due to multiple organ failure, and two who died during follow-up (one due to acute myocardial infarction and one due to severe pulmonary infection). LSA stent occlusion was observed in one patient in the CSG group at 18 months postoperatively, likely due to compression by the metal wires of the thoracic aortic stent graft at the fenestration site.

**Figure 3:**
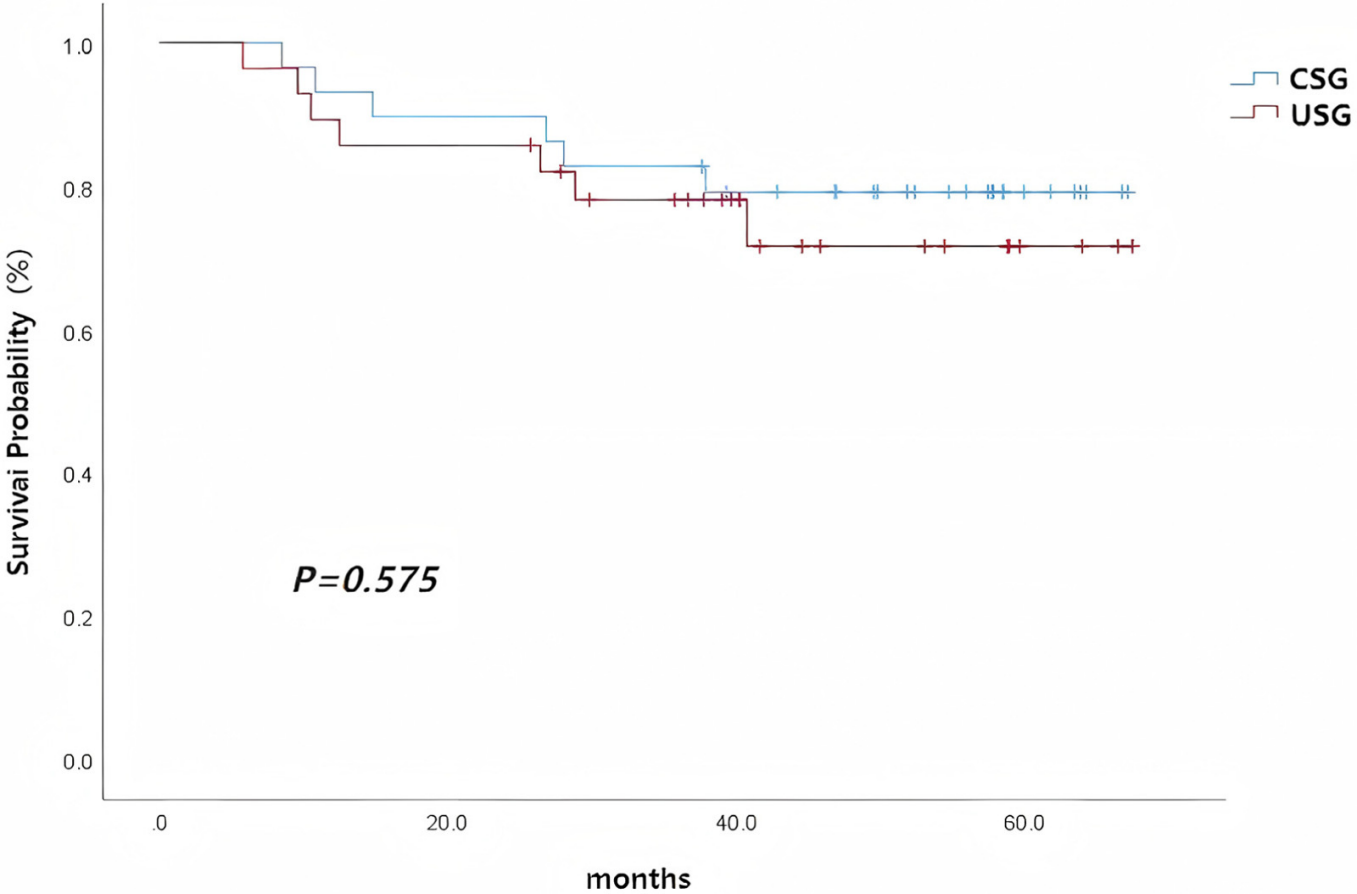
Overall survival curves of the combined surgery group and the uncombined surgery group in patients with Stanford type A aortic dissection.

In the uncombined surgery group, two patients were lost to follow-up, with a follow-up rate of 92.7%. 21 of 28 patients (75.0%) achieved successful LSA bypass grafting at surgery. Among these 21 patients, the bypass graft patency rate was 100% (21/21) at discharge. Among the remaining patients, five died within 5 years, including two who died in-hospital (one due to pulmonary infection leading to multiple organ failure and one due to heart failure), and three who died during follow-up (two due to severe pulmonary infection and one with an unknown cause of death). Among all patients who successfully underwent LSA reconstruction in the USG, a graft thrombus accompanied by stenosis was identified in one patient in the USG group at 9 months postoperatively, which was considered to be caused by graft kinking. The patient remained asymptomatic and did not require surgical intervention. Kaplan–Meier survival analysis showed no difference in 5-year survival rates between the combined surgery group and the uncombined surgery group (*χ*^2^ = 0.315, *P* = 0.575, Figure 3; Table 4).

**Table 4:** Number at risk.

Group	Baseline (0y)	1 Year (1y)	3 Years (3y)	5 Years (5y)
CSG’	29	27	24	23
USG	28	25	23	21

## 5 Discussion

### 5.1 Advantages of *in situ* Laser fenestration for LSA reconstruction

Although some studies have shown that carotid-subclavian transposition and carotid-subclavian bypass grafting achieve good long-term patency rates and acceptable complication rates while preserving the LSA (8–10), these techniques require extensive dissection and exposure of the LSA. The LSA is often deep and difficult to expose and anastomose, posing risks of recurrent laryngeal nerve and vascular injury during surgery, and prolonging operative time (5). Simplifying the surgical procedure and minimizing operative and CPB times are crucial to improving patient outcomes.

Some studies suggest that covering the LSA is safe in patients undergoing thoracic endovascular aortic repair who are in critical condition or have difficult *in situ* reconstruction (11–13). Moreover, some scholars believe that in patients with Stanford type A dissection, if intraoperative exposure is difficult, the LSA can be ligated after strict evaluation of collateral circulation (14, 15). However, evaluating collateral circulation in acute Stanford type A aortic dissection patients is challenging and there is no universally accepted quantitative indicator. Additionally, LSA ligation increases the risk of spinal cord ischemia or paraplegia (16), especially in patients who may require more than one aortic reconstruction surgery, for whom maintaining adequate spinal perfusion is essential to prevent paraplegia. Furthermore, direct surgical intervention on the LSA is associated with a higher risk of recurrent laryngeal nerve injury (17). Preserving the LSA is important for patients who have undergone or may undergo left internal mammary artery-coronary artery or left axillary artery-femoral artery bypass grafting. For left-handed patients, maintaining LSA patency is essential for normal left upper limb function. The Society for Vascular Surgery guidelines strongly recommend LSA revascularization in patients who may require the left internal mammary artery for coronary artery bypass grafting, have a dominant left vertebral artery, have a left arm dialysis fistula, or require long-segment coverage of the descending thoracic aorta (≥20 cm) that may compromise multiple intercostal arteries (18). For some Stanford type A aortic dissection patients who require visceral artery reconstruction, especially those requiring reconstruction of the four visceral branches, preserving the physiological anatomy of the LSA provides an important access route for the surgery. LSA reconstruction can be performed before or after vascular intervention (19).

In this study, we used *in situ* laser fenestration of the LSA. Compared with open surgery, *in situ* laser fenestration is faster, simpler, safer, and is associated with a higher success rate, a lower technical threshold, fewer perioperative complications, and higher mid-term patency rates (20). Our results show that *in situ* laser reconstruction shortens operative time, reduces the incidence of nerve injury and other complications, and has satisfactory short-term patient outcomes. In the uncombined surgery group, LSA reconstruction failed in six patients, and the LSA was ligated. In five of these cases, the LSA was deep and difficult to expose and reconstruct, and the surgeon judged that even if anastomosis was successful, there was a high risk of nerve injury or posterior wall bleeding. In one case, the ascending aorta was aneurysmal, and the LSA was displaced and difficult to expose.

### 5.2 Challenges and considerations when performing the *in situ* laser fenestration technique

Several challenges arise when performing *in situ* laser fenestration. First, tortuous vessel anatomy or a small angle between the branch vessel and the aortic arch can increase the difficulty of fenestration. In such cases, the laser fiber may shift relative to the aortic stent graft. A steerable sheath or multi-purpose catheter can be used to adjust the angle between the LSA and the main stent graft to improve fenestration success rates.

Second, before fenestration, right anterior oblique and axial aortic angiography should be performed to confirm that the sheath tip is perpendicular to the aortic stent graft.

Third, fenestration may cause the main stent graft to shift. Therefore, forceful balloon passage through the fenestration should be avoided. A balloon with a fine tip and good tracking should be selected and the initial balloon diameter should be more than 4 mm, with gradual dilation. The appearance of a “waist” during balloon dilation helps confirm successful fenestration.

Fourth, assistant cooperation is crucial during fenestration. The assistant must keep the balloon and sheath positions relatively fixed to facilitate selection of the fenestration site. The branch stent is usually deployed using a “parachute” technique, avoiding excessive protrusion into the aortic stent graft or covering the vertebral artery.

The surgeon must thus have extensive experience in endovascular treatment of Stanford type B aortic dissection, be proficient in selecting aortic stent graft sizes and deployment techniques, and be familiar with the laser fenestration process and precautions to shorten operative time and reduce perioperative complications. Additionally, if the LSA is tortuous or has a small angle with the aorta, fenestration may fail, and therefore these factors should be taken into consideration when choosing *in situ* laser fenestration. Furthermore, in patients with long-segment LSA dissection, *in situ* laser fenestration may be contraindicated due to the risk of postoperative LSA false lumen flow and type II endoleak. The reported incidence of endoleaks following laser *in situ* fenestration during TEVAR procedures is approximately 4.7% in the literature (21). In the present study, no significant postoperative endoleaks were observed, which may be attributed to stringent patient selection criteria. Therefore, we recommend that the feasibility and safety of *in situ* laser fenestration should be carefully evaluated by experienced vascular surgeons prior to the procedure.

In situ laser fenestration of the LSA is generally chosen for patients with difficult LSA exposure, reconstruction, or hemostasis. Because this technique increases medical costs and requires long-term postoperative antiplatelet therapy, we communicate with the families of patients who are preoperatively predicted to have difficult LSA reconstruction (especially obese patients) and decide whether to perform *in situ* laser fenestration based on the family's wishes. Typically, the LSA is deeper and harder to expose and anastomose in obese patients, and thus we recommend prioritizing *in situ* laser fenestration for patients with a BMI ≥28 kg/m^2^.

### 5.3 Feasibility and generalizability considerations

The requirement for a hybrid operating room represents a significant limitation for widespread adoption of this technique. Hybrid suites combine advanced imaging capabilities (digital subtraction angiography) and conventional surgical infrastructure, which are currently available only in specialized centers. While only 22% of Chinese tertiary hospitals currently have hybrid ORs (2023 National Health Commission report), Increasing government investment in hybrid surgical platforms and cost-sharing models through regional referral networks may improve accessibility.

### 5.4 Study limitations

This study has several limitations. First, the study population is small and the follow-up time is limited. Second, owing to the small number of patients with reconstruction failure, multivariate logistic regression analysis of reconstruction failure was not performed; therefore, our conclusions need to be validated by large-sample randomized controlled trials. Finally, patients were not randomly assigned into groups, which may have introduced some selection bias.

### 5.5 Limitations of bypass patency

Although our cohort showed 100% early bypass patency, this does not reflect long-term durability. A recent study reported 91.4% 5-year patency for extra-anatomical LSA bypass (22), suggesting our mid-term patency data (95.2% at 5 years) aligns with literature. Continuous surveillance is critical for detecting late graft degeneration.

## 6 Conclusion

In situ laser reconstruction of the LSA combined with aortic arch debranching significantly shortens operative time, reduces the incidence of nerve injury and other complications, and has satisfactory short-term follow-up outcomes in patients with acute Stanford type A aortic dissection. This technique provides a new surgical option for patients with difficult LSA reconstruction.

## Data Availability

The original contributions presented in the study are included in the article/Supplementary Material, further inquiries can be directed to the corresponding authors.
